# Langerhans Cell Histiocytosis Mimicking Osteomyelitis in an Infant

**Published:** 2013-07-09

**Authors:** Havva Erdem, Nilüfer Kadıoğlu, Ali Kemal Uzunlar, İstemi Yücel, Murat Oktay, Fahri Halit Beşir

**Affiliations:** Department of Pathology, Duzce University of Medical Faculty, Duzce, 81000, Turkey.; Department of Pathology, Duzce University of Medical Faculty, Duzce, 81000, Turkey; Department of Pathology, Duzce University of Medical Faculty, Duzce, 81000, Turkey; Department of Orthopedics, Duzce University of Medical Faculty, Duzce, 81000, Turkey; Department of Pathology, Duzce University of Medical Faculty, Duzce, 81000, Turkey; Department of Radiology, Duzce University of Medical Faculty, Duzce, 81000, Turkey

**Dear Sir,**

 Langerhans cell histiocytosis (LCH) is a relatively rare disorder, accounting for less than 1% of all osseous lesions. LCH involving bone has been reported in a wide age distribution ranging from the first few months to the 8th decade of life.[1] Pain and swelling of the affected area occur most commonly. Other findings are related to the bone involved. Clinically and imaging studies can point out malignancy or infection.[2] Herein we report a case of Langerhans cell histiocystosis of femeur which simulated as osteomyelitis.

A 9-month-old male infant was referred to our hospital with right hip pain for 10 days. Clinical examination showed a soft mobile palpable mass on greater trochanter of right femur. Radiograph showed radiolusent lytic lesion that was localized on the upper metaphysis of right femur with periosteal reaction and little expansion. Adjacent soft tissues showed increased density and thickness (Fig. 1). Magnetic resonance imaging (MRI) revealed a heterogeneous contrasting lesion on upper right femur that was hypointense on T1 and hyperintense on T2 weighted images. Also there was a 5 mm x 2 mm area on his right distal femur which was hyperintense on T2, and hypointense on T2 weighted images. ESR was 69 mm first hour and hemoglobin 11.1 g/dl. Other laboratory tests were within normal limits. Patient was shifted to orthopedics department. Clinically the case was considered as osteomyelitis and after optimisation, operation was performed. Simple curettage of inflammed area of the bone was done. Removed tissue was sent for histopathology.

**Figure:**
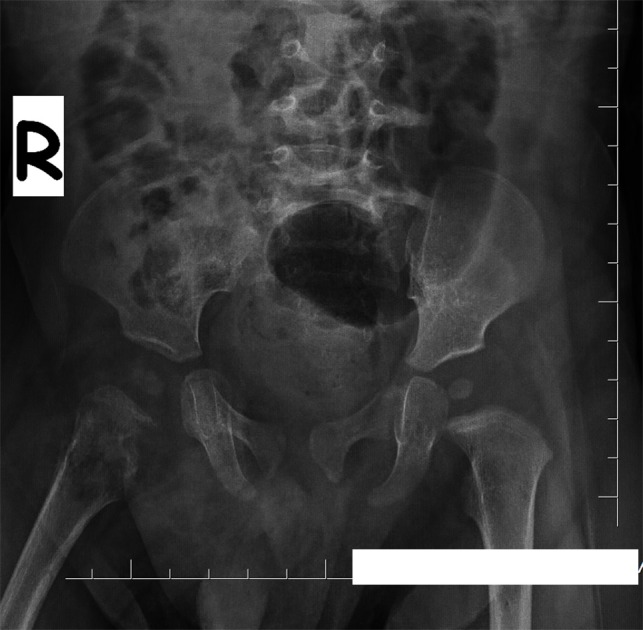
Figure 1: Magnetic resonance imaging (MRI) revealed a heterogeneous contrasting lesion on upper right femur: hypointense on T1 and hyperintense on T2 weighted images

Grossly, there were 3 pieces of material (0.7cm to 2 cm in size) with a yellowish-pinky colour. Microscopically there were numerous bean shaped atypical histiocytes, eosinophils and neutrophils (Fig. 2). Immunohistochemically, these atypical histiocytes were CD1A, S100,CD3 positive (Fig. 3). The case was diagnosed as Langerhans cell histiocytosis. The patient is doing fine at biannuall follow-ups. Clinically and radiographically there are no signs of recurrence.

**Figure:**
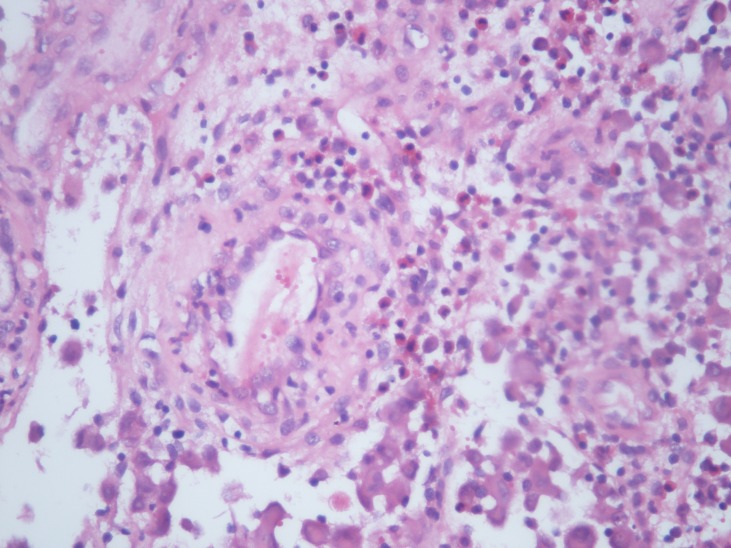
Figure 2:Numerous eosinophils. X400 H/E

**Figure:**
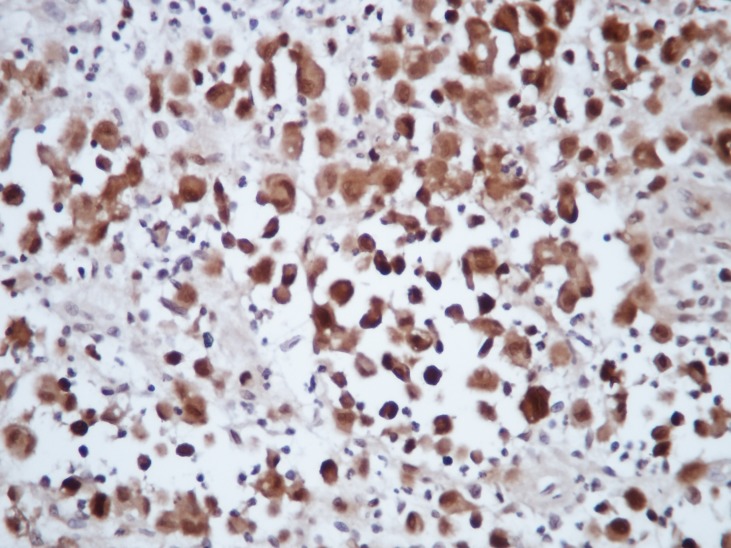
Figure 3: Atypic histiocytes showing strong S100 positivity

The case was diagnosed as Langerhans cell histiocytosis. The patient is doing fine at biannuall follow-ups. Clinically and radiographically there are no signs of recurrence. LCH of bone can be divided into three major categories on the basis of type and extent of the organ involvement: solitary bone involvement, multiple bone involvement (with or without skin involvement), multiple organ involvement (bone, liver, spleen, and others).[3] The most common sites are the cranial vault, jaw, humerus, rib, and femur. In our case femur was involved. The classical cytological features include high cellularity composed of sheets and isolated LCH seen admixed with polymorphous population of numerous eosinophils, neutrophils, lymphocytes, plasma cells, multinucleated giant cells, and macrophages. They show variable degree of pleomorphism and mitotic activity. The key to the diagnosis is to identify the LCH through its characteristic features, namely, nuclear grooves and nuclear pseudoinclusions. Presence of dendrite-like cytoplasmic processes in LCH is a rare but is a characteristic feature. We observed many atypical histiocytes but could not identify any demonstrative cytoplasmic process. Degree of eosinophil infiltration varies in different areas of LCH lesion and different organs, thus their number can vary from scant to abundant in cytology smears. Their presence can help in diagnosis.[4]

Histopathologically, there are dense intradermal infiltrations of histiocytes showing abundant eosinophilic cytoplasm and kidney-shaped or indented nuclei associated with lymphocytes and eosinophils. These histiocytes show strong expression of S-100 protein and CD1a, markers of LCs. Differential diagnosis includes hemangioma, juvenile xanthogranuloma, Spitz nevi, mastocytoma, osteoid osteoma, osteoblastoma , broad and includes infection, malignancies such as Ewing sarcoma, lymphoma, or metastasis.[5-7] LCH has a lytic appearence with an ill defined border in the imaging studies similar to osteomyelitis so osteomyelitis must be kept in differantial diagnosis.[8]

In literature there are different options for managing LCH. First option is not to do anything, because it can spontaneously regress. Operation can be performed for either to make a diagnosis or to relieve symptoms by partial resection. Topical or intralesionel steroids can be used. Radiotherapy is another option. Chemotherapy can be used in systemic cases. Recurrences usually appear in 2 years but duration can be very long so it is important to follow these patients for a long period of time with imaging studies.[9] In our case simple curettage was performed. After the operation patient's mobility improved. His serial imaging studies showed no sign of recurrence, and to date he is healthy.

## Footnotes

**Source of Support:** Nil

**Conflict of Interest:** None declared

